# Lower serum calcium is independently associated with CKD progression

**DOI:** 10.1038/s41598-018-23500-5

**Published:** 2018-03-26

**Authors:** Cynthia J. Janmaat, Merel van Diepen, Alessandro Gasparini, Marie Evans, Abdul Rashid Qureshi, Johan Ärnlöv, Peter Barany, Carl-Gustaf Elinder, Joris I. Rotmans, Marc Vervloet, Friedo W. Dekker, Juan Jesus Carrero

**Affiliations:** 1 Department of Clinical Epidemiology, Leiden University Medical Center, Leiden, The Netherlands; 2 Department of Clinical Science, Karolinska Institutet, Stockholm, Sweden; 3 Public Healthcare Services committee, Stockholm County Council, Stockholm, Sweden; 4 Department of Nephrology, Leiden University Medical Center, Leiden, The Netherlands; 5 Department of Nephrology and Institute for Cardiovascular Research VU, VU University Medical Center, Amsterdam, The Netherlands; 6 Department of Medical Epidemiology and Biostatistics, Karolinska Institutet, Stockholm, Sweden

## Abstract

Disturbances in calcium metabolism are common in individuals with chronic kidney disease (CKD), but whether they are associated with subsequent kidney function decline is less clear. In a CKD 3–5 cohort of 15,755 adult citizens of Stockholm with creatinine tests taken during 2006–2011 and concurrent calcium testing at cohort entry, we investigated the association between baseline serum calcium and the subsequent change in estimated glomerular filtration rate (eGFR, by CKD-EPI) decline using linear mixed models. Mean (SD) baseline corrected serum calcium was 9.6 (0.5) mg/dL. Mean (95%-confidence interval [CI]) eGFR decline was −0.82 (−0.90; −0.74) mL/min/1.73 m^2^/year. In advanced CKD stages, higher baseline serum calcium was associated with less rapid kidney function decline. The adjusted change (95%-CI) in eGFR decline associated with each mg/dL increase in baseline serum calcium was −0.10 (−0.28; 0.26), 0.39 (0.07; 0.71), 0.34 (−0.02; 0.70) and 0.68 (0.36; 1.00) mL/min/1.73 m^2^/year for individuals in CKD stage 3a, 3b, 4, and 5, respectively. In a subgroup of patients using vitamin D supplements, the association between baseline serum calcium and CKD progression was eliminated, especially in CKD stage 3b and 4. To conclude, in individuals with CKD stage 3b to 5, lower baseline corrected serum calcium, rather than higher baseline serum calcium, associated with a more rapid CKD progression. Lower serum corrected calcium seems to be indicative for vitamin D deficiency.

## Introduction

The identification of modifiable risk factors for chronic kidney disease (CKD) progression is important to the design, study and implementation of preventive strategies^1,2^. Disturbances in mineral metabolism are prevalent in advanced CKD stages and have been suggested not only to be the consequence of CKD, but also a potential cause for a more rapid kidney function decline^3,4^. Hyperphosphatemia has been consistently associated with CKD progression^5–7^, as well as FGF-23 excess and the calcium-phosphorus product^8,9^. Less evidence exists on the association between calcium disturbances and kidney function decline, with two recent studies reporting conflicting and counterintuitive associations: while Schwarz *et al.*^8^ found no association between calcium and CKD progression in CKD stage 1–5 patients, Lim *et al.*^10^ reported low serum calcium to be associated with a faster kidney function decline in a pooled cohort of CKD stage 3–4 patients. Intuitively, it would be expected that high serum calcium concentrations contribute to rapid kidney function deterioration, due to precipitation of calcium-phosphorus product in vessels causing vascular calcifications^11^, or to acute effects of hypercalcemia. Preceding studies used a composite outcome of progression (50% decline or eGFR slope >−5 mL/min/1.73 m^2^ plus initiation of renal replacement therapy [RRT]), and did not investigate the absolute change in kidney function for each CKD stage. Furthermore, the kidney has compensatory mechanisms to maintain calcium-phosphate balance until late CKD stages^12,13^, and therefore serum calcium may solely appear as overt risk factor for progression in advanced CKD^12^. To clarify this issue, we here aimed to determine the plausible association between serum calcium and subsequent kidney function decline in non-dialysis patients with CKD stages 3–5 separately from a large regional-representative healthcare system.

## Results

### Baseline characteristics

**Figure 1:**
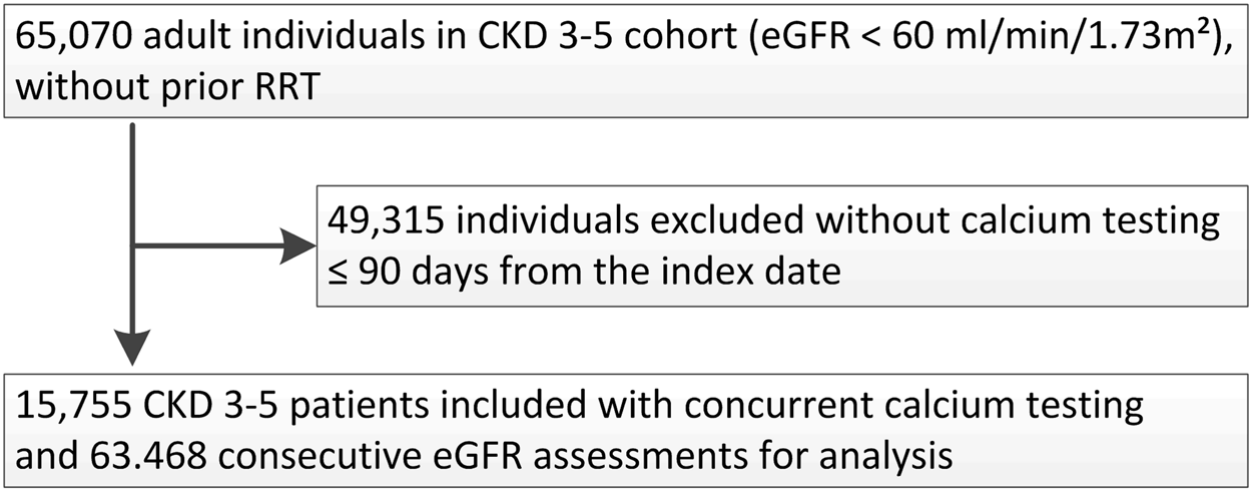
Flowchart of patient inclusion.

Out of a total of 65,070 adult individuals with an eGFR at study entry that qualified as CKD 3–5, we included 15,755 for whom concurrent calcium was measured. See Fig. 1 for a flowchart of patient inclusion. These patients had a total of 63,468 consecutive eGFR assessments during observation. Median (IQR) age was 79.9 (70.2–85.8) years, and 39% were men. Median (IQR) eGFR was 48.1 (37.2–55.0) mL/min/1.73 m^2^. A total of 9,286 patients had CKD stage 3a, 4,190 patients had CKD stage 3b, 1,784 patients had CKD stage 4 and 495 patients had CKD stage 5. Baseline characteristics are shown in Table 1. The majority of participants had baseline corrected calcium levels within the normal reference range, i.e. 8.6–10.2 mg/dL (2.15–2.55 mmol/L)^14^. Only 1.1% and 7.4% of participants had hypo- and hypercalcemia, respectively. In participants with hypocalcemia, 30% received vitamin D therapy, and only one person received active vitamin D therapy. Participants with CKD stage 5 were younger and more often men than the patients with CKD stages 3a to 4. Diabetes mellitus, hypertension, albuminuria and hyperphosphatemia were more prevalent in CKD stage 5 compared to other CKD stages. CKD stage 5 participants used phosphate binders more often than other CKD stages, and those with CKD stages 4–5 more often used active vitamin D analogues and diuretics compared to stage 3. Twelve variables were used as potential confounders and used to impute missing values. Ten of these variables were complete in all patients. Hemoglobin and phosphorus, had 15% and 71% of missings, respectively. As anticipated from a healthcare extraction, a few participants had a dipstick albuminuria or an iPTH test taken at the index date. Because these variables were available for 13% and 8% of the total study population, respectively, they were not considered for multivariable adjustment in our primary analysis.

**Table 1:** Baseline characteristics of the study population by CKD stage^a^.

	All (*n* = 15,755)	CKD 3a *(n* = 9,286)	CKD 3b (*n* = 4,190)	CKD 4 (*n* = 1,784)	CKD 5 (*n* = 495)
**Age (years)**	79.9 (70.2–85.8)	79.0 (69.8–85.1)	81.9 (73.5–87.2)	80.1 (68.2–86.4)	73.2 (61.6–82.4)
**Sex (% men)**	6,140 (39.0)	3,323 (35.8)	1,676 (40.0)	841 (47.1)	300 (60.6)
**Comorbidities (%)** ^b^					
Diabetes mellitus	2,352 (14.9)	1,012 (10.9)	723 (17.3)	480 (26.9)	137 (27.7)
Cardiovascular disease	1,502 (9.5)	722 (7.8)	479 (11.4)	251 (14.1)	50 (10.1)
Hypertension	9,794 (62.2)	5,035 (54.4)	2,981 (71.1)	1,411 (79.1)	399 (80.6)
Corrected calcium (mg/dl)^∗^	9.5 ± 0.5	9.5 ± 0.5	9.6 ± 0.5	9.5 ± 0.6	9.6 ± 0.9
Hypercalcemia (>10.2 mg/dl)	1,165 (7.4)	560 (6.0)	371 (8.9)	161 (9.0)	73 (14.7)
Hypocalcemia (<8.6 mg/dl)	179 (1.1)	63 (0.7)	34 (0.8)	46 (2.6)	36 (7.3)
*Vitamin D use (%)* ^*c*^	*55 (30.7)*	*4 (6.3)*	*6 (17.6)*	*19 (41.3)*	26 (72.2)
*Active vitamin D use (%)* ^*c*^	*1 (0.6)*	*0 (0.0)*	*0 (0.0)*	*0 (0.0)*	1 (2.8)
**Albumin corrected calcium (mg/dl)** ^**∗**^	9.6 ± 0.5	9.6 ± 0.5	9.6 ± 0.5	9.6 ± 0.6	9.7 ± 0.8
**Serum albumin (g/l)** ^**∗**^	37.0 ± 4.1	37.5 ± 3.8	36.6 ± 4.1	35.8 ± 4.5	35.4 ± 4.8
**Albuminuria (% yes)** ^**d**^	720 (4.6)	270 (2.9)	205 (4.9)	167 (9.4)	78 (15.8)
**Baseline eGFR (ml/min/1.73 m** ^**2**^ **)**	48.1 (37.2–55.0)	53.9 (49.9–57.2)	38.8 (34.9–42.2)	24.4 (20.3–27.5)	11.0 (8.5–13.1)
**Number of repeated eGFR tests**	5.0 (2.0–13.0)	4.0 (1.0–10.0)	6.0 (2.0–13.0)	9.0 (4.0–17.0)	8.0 (4.0–15.8)
**Phosphorus (mg/dl)** ^**∗**^	3.7 ± 0.9	3.4 ± 0.6	3.5 ± 0.7	3.9 ± 0.8	5.1 ± 1.3
iPTH (pg/ml)*	143.8 ± 144.6	72.8 ± 49.1	104.0 ± 67.5	161.1 ± 125.5	270.2 ± 249.5
**Serum Hb (g/l)**	131.5 ± 16.0	134.7 ± 15.2	129.2 ± 15.7	123.8 ± 15.7	119.1 ± 16.5
**Medication (%)**					
Calcium supplements	105 (0.7)	88 (0.9)	15 (0.4)	1 (0.1)	1 (0.2)
Bisphosphonates	836 (5.3)	537 (5.8)	240 (5.7)	51 (2.9)	8 (1.6)
Phosphate binders	313 (2.0)	9 (0.1)	40 (1.0)	112 (6.3)	152 (30.7)
Vitamin D therapy	915 (5.8)	99 (1.1)	175 (4.3)	366 (20.5)	275 (55.6)
*Active vitamin D use*	*54 (0.3)*	*10 (0.1)*	*16 (0.4)*	*18 (1.0)*	10 (2.0)
Diuretics	7,876 (50.0)	3,842 (41.4)	2,440 (58.2)	1,252 (70.2)	342 (69.1)
*Thiazide diuretics*	*492 (3.1)*	*335 (3.6)*	*124 (3.0)*	*31 (1.7)*	*2 (0.4)*
*Loop diuretics*	*2,184 (13.9)*	*995 (10.7)*	*689 (16.4)*	*410 (23.0)*	*90 (18.2)*

^a^Continuous variables are expressed as mean ± standard deviation or median (interquartile range), and categorical variables are expressed as number (percentage).

^b^Comorbidities are deduced from Charlson domains. ^c^These numbers only apply to patients with hypocalcemia (<8.6 mg/dL).

^d^% Albuminuria is presented as percentage of the total study population, instead of the percentage of the patient population in which an actual albuminuria test was performed. Due to the missingness, the percentages shown are an underestimation of the actual percentage of albuminuria in the study population.

*To convert serum albumin in g/dl to g/l, multiply by 10; serum calcium in mg/dl to mmol/l, multiply by 0.2495; serum phosphorus in mg/dl to mmol/l, multiply by 0.3229; serum iPTH in pg/ml to ng/l, multiply by 1.

### Association between baseline serum calcium and subsequent kidney function decline

**Figure 2:**
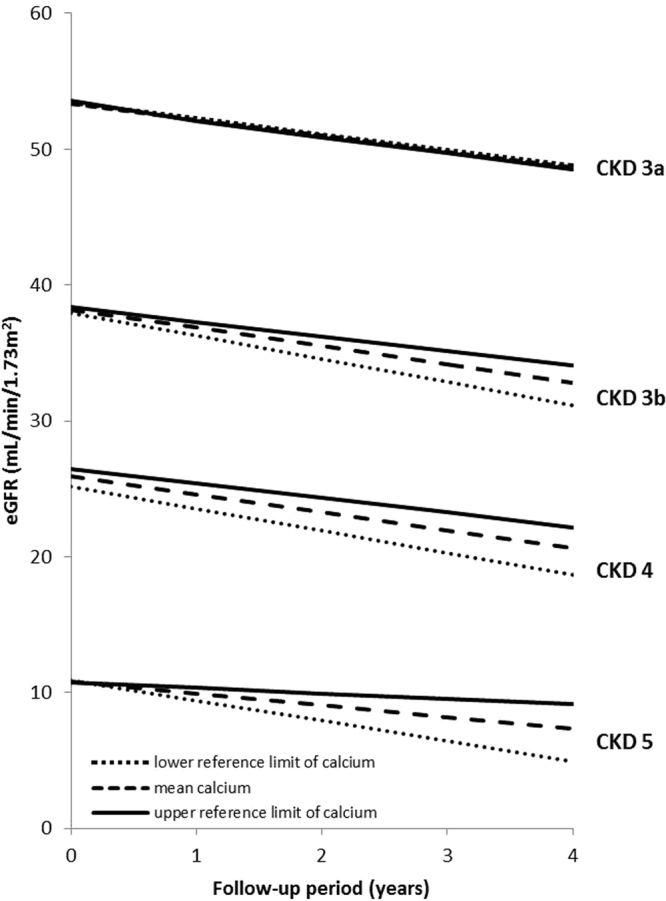
Modelled longitudinal trajectories in eGFR associated with corrected baseline serum calcium levels in CKD stage 3a, 3b, 4, and 5. Provided are the calcium GFR trajectories based on the fully adjusted linear mixed model for the overall mean corrected baseline calcium level, the lower (8.6 mg/dL) and upper (10.2 mg/dL) reference limits, assuming the mean for continuous covariates and the mode (most frequent values) for categorical covariates the study population in each CKD stage.

The median (IQR) length of follow-up was 4.3 (2.0–5.3) years, and the median (IQR) number of eGFR measurements per patient was 5.0 (2.0–13.0). The overall mean annual rate of decline in patients with CKD stages 3a-5 was −0.82 (95% CI −0.903; −0.738) mL/min/1.73 m^2^, and the mean annual rate of decline was −0.657 (95% CI −0.775; −0.539), −1.013 (95% CI −1.175; −0.851), −1.457 (95% CI −1.634; −1.279) and −0.965 (95% CI −1.294; −0.636) mL/min/1.73 m^2^ for patients with CKD stage 3a, 3b, 4 and 5, respectively. The (adjusted) change in the rate of decline in kidney function associated with one unit higher (i.e. mg/dl) of serum calcium is shown in Table 2. While no association was observed between serum calcium at baseline and subsequent eGFR decline in patients with CKD stage 3a, a consistent negative association was found in the remaining CKD stages: in other words, for every unit higher in baseline serum calcium, the associated eGFR decline was slower. The other way around, lower baseline serum calcium is associated with a faster subsequent kidney function decline. The adjusted associations in these stages are substantial, ranging from an increase of 24% to 70% of the mean annual decline rate for every unit lower in serum calcium. Aforementioned is illustrated in Fig. 2, which shows the modelled longitudinal trajectories in eGFR associated with corrected baseline serum calcium levels in CKD stage 3a to 5. Provided in the figure are the calcium eGFR trajectories based on the fully adjusted linear mixed model for the mean corrected baseline calcium level per CKD stage, the lower (8.6 mg/dL) and upper (10.2 mg/dL) reference limits, assuming the mean and the mode from the study population in each CKD stage for continuous and categorical covariates, respectively. Furthermore, a dose-response relationship seemed present: for higher CKD stages, lower serum calcium was associated with a more rapid kidney function decline, i.e. the lower the eGFR, the stronger the effect of lower calcium on subsequent decline (Table 2). This was confirmed by multiplicative interaction tests between baseline eGFR and serum calcium (Table 3). The negative interaction term indicates a smaller coefficient for higher eGFR. Let us suppose the adjusted value of 0.019 mL/min/1.73 m^2^: this means given that we have one unit increase in baseline eGFR, one unit increase in baseline calcium results in a smaller additional change in eGFR decline of 0.019 mL/min/1.73 m^2^. In other words, the effect of serum calcium on kidney function decline is stronger, for lower baseline eGFR, thus the higher the CKD stage.

**Table 2:** Association between baseline corrected serum calcium and the subsequent rate of kidney function decline (95%-CI).

	CKD 3a *(n* = 9,286)	P*	CKD 3b (*n* = 4,190)	P*	CKD 4 (*n* = 1,784)	P*	CKD 5 (*n* = 495)	P*
Change in eGFR decline per each mg/dL higher albumin-corrected calcium (negative = extra decline)^a^								
Raw data	−0.098 (−0.362; 0.165)	0.46	0.515 (0.196; 0.835)	0.002	0.428 (0.085; 0.772)	0.01	0.649 (0.323; 0.975)	<0.001
Model 1	−0.003 (−0.044; 0.038)	0.98	0.390 (0.073; 0.707)	0.02	0.328 (−0.003; 0.686)	0.07	0.683 (0.359; 1.008)	<0.001
Model 2	−0.009 (−0.277; 0.260)	0.95	0.391 (0.074; 0.708)	0.02	0.344 (−0.015; 0.704)	0.06	0.682 (0.355; 1.009)	<0.001

^a^In mL/min/1.73 m^2^ per year.

Model 1 adjusted for age, sex, blood pressure, DM, CVD, serum albumin and hemoglobin.

Model 2 adjusted for covariates in model 1 plus serum phosphorus, active vitamin D therapy and calcium supplements.

*P-value for difference in the change in the rate of kidney function decline with one unit higher serum calcium.

**Table 3:** Multiplicative interaction tests between baseline corrected serum calcium and baseline eGFR in its association with subsequent kidney function decline (95%-CI).

	All patients (n = 15,755)	P*
Additional change in eGFR decline per each mg/dL higher albumin-corrected calcium for each mL/min/1.73 m^2^ higher unit of eGFR (negative = smaller effect)		
Raw data	−0.021 (−0.032; −0.009)	<0.001
Model 1	−0.019 (−0.030; −0.008)	0.001
Model 2	−0.019 (−0.030; −0.008)	0.001

Model 1 adjusted for age, sex, blood pressure, DM, CVD serum albumin and hemoglobin. Model 2 adjusted for covariates in model 1 plus serum phosphorus, active vitamin D therapy and calcium supplements. *P-value for difference in the change in the rate of kidney function decline with one unit higher serum calcium.

### Sensitivity analyses

Various sensitivity analyses were performed. (1) Additional adjustment for baseline eGFR values yielded similar results (Supplementary Table S1). (2) A subgroup analysis in patients with vitamin D supplementation at baseline showed that the association between baseline corrected serum calcium and subsequent kidney disease progression is abrogated among users of vitamin D medication (Supplementary Table S2). (3) To test the possible impact of albuminuria and iPTH adjustment, we performed multiple imputation analysis on these covariates and observed comparable results in our models (Supplementary Tables S3a-b). (4) Repeating the main analyses with separate adjustment for diuretics and hypertension yielded similar results (Supplementary Table S4). (5) Trend analysis in each CKD stage by quintiles of serum calcium distribution, suggested a gradual (and not a non-linear) higher rate of eGFR decline with lower serum calcium at baseline, in particular for patients with CKD stage 5 (Supplementary Table S5). (6) The magnitude of the association was confirmed when using uncorrected serum calcium (Supplementary Tables S6a-b). (7) Similar results were obtained when repeating the analysis in patients with serum corrected calcium levels within the normal reference range (Supplementary Tables S7a-b). (8) The results were similar when selecting individuals with at least 3 eGFR tests available (Supplementary Tables S8a-b). (9) We observed similar associations in the complete case analysis (without imputation) (Supplementary Tables S9a-b). (10) Finally, we tested the association between calcium and time to event analysis for dichotomous endpoints of CKD progression. In total, 629 (4%) patients started RRT, 1594 (10%) had a sustained GFR decline of more than 30% and 5436 (35%) died during follow-up. In the adjusted Cox proportional-hazards regression analysis, a borderline not significant lower risk of a sustained GFR decline of >30% was present for each mg/dL increase in baseline corrected calcium levels, for both CKD stage 4 and 5. This association was not present in CKD stage 3a and 3b (Supplementary Table S10). In addition, adjusted Cox proportional-hazards regression analysis showed a trend towards higher risk of RRT with lower calcium levels at baseline (Supplementary Table S11). Although, this association was only significant for CKD stage 4, the observed trend is consistent with findings obtained from linear mixed models.

## Discussion

Intuitively, a higher serum calcium would be expected to be associated with a more rapid kidney function deterioration^11^. In contrast, we demonstrate in this study that lower baseline serum calcium, already within the normal reference range, is associated with a subsequent more rapid eGFR decline in individuals with CKD stages 3b-5. We showed that the adjusted change in kidney function decline was attenuated by a value between 0.34 and 0.68 mL/min/1.73 m^2^ for CKD stages 3b to 5, which corresponds to 24–70% reduction of the mean annual decline rate, for every unit increase in calcium. Thus, the effects are potentially large, especially considering that serum calcium can easily vary between 9 and 10 mg/dL in these patients. This observation confirms and expands previous literature and underscores the need for a better understanding of the role of calcium in CKD progression^8,10^. Strengths of our analysis are its large, real-world healthcare setting, the study of kidney function decline rate, and the *a priori* separation of CKD stages, allowing weighing the relative contribution of calcium to CKD progression rate for each CKD stage^12,13^.

Our observational study does not allow inference of causality in the association between serum calcium and CKD progression. Our results are similar to those of Taylor *et al*., who showed that a low, rather than high, urinary calcium excretion associated with increased risk of CKD^15^. Current knowledge of the pathophysiology of CKD-MBD favors the argument of lower calcium being a risk marker and/or proxy of other underlying processes: in the natural history of (untreated) CKD progression, hypocalcemia usually develops and is associated with secondary hyperparathyroidism^16^. Physiologically, 1,25-dihydroxyvitamin D3 (1,25(OH)_2_D3) enhances intestinal calcium absorption. Since declining of 25(OH)D3 and especially 1,25(OH)_2_D3 is an early feature of CKD, hypocalcemia in CKD is generally considered to be a consequence of that^12^. Low levels of the 25(OH)D3 substrate may contribute to decreased levels of 1,25(OH)_2_D3 production, particularly in CKD patients with nephrotic range proteinuria^12^. Therefore, it is possible that a lower serum calcium in this setting might indicate suboptimal supplementation of vitamin D deficiency, assuming a pathophysiological role in CKD progression of vitamin D deficiency. Both experimental and epidemiologic studies have shown that 25(OH)D3 deficiency itself might contribute to a progressive decline in kidney function^17–19^. In a subgroup analysis in patients using vitamin D supplements at baseline, we observed that the association between baseline serum calcium and subsequent kidney disease progression was abbrogated in participants with CKD stage 3b and 4. This supports the hypothesis that a lower serum corrected calcium at baseline may be indicative for vitamin D deficiency. Also, in CKD stage 5 the association between lower serum calcium concentrations and CKD progression was attenuated among vitamin D users, although not abbrogated. This might indicate suboptimal supplementation of native vitamin D in this patient group, which indeed in general has the highest dose requirements. In addition to the role of 25(OH)D3, the impaired kidney function in CKD patients results in limited capacity to produce 1,25(OH)_2_D3 out of 25(OH)D3, due to the smaller amount of 1α-hydroxylase. Because of the low prevalence of active vitamin D use in our study population (sampled shortly before this medication entered in the Swedish market), correcting for active vitamin D therapy did not influence our results and the results should be interpreted with caution. Recently, low 1,25(OH)_2_D3 levels has been attributed to FGF23 accumulation^20,21^. In turn, elevated levels of FGF-23 have been consistently associated with CKD progression^22,23^ and could in itself be a risk factor for kidney function decline via increased phosphate excretion per nephron, not mediated by 1,25(OH)_2_D3^9,24^. Furthermore, Jean *et al*. showed that the use of oral cholecalciferol corrected vitamin D deficiency in dialysis patients, thereby also increasing the level of serum 1,25(OH)_2_D3 threefold^25^. Altogether, we speculate that mainly decreased vitamin D concentrations and associated suboptimal native vitamin D supplementation, and/or elevated FGF23, explain the association between lower serum calcium and CKD progression observed in CKD stages 3b to 5. This remains an observational study and in any case, the finding that lower serum calcium increases the rate of kidney function decline needs confirmation and further exploration in experimental studies.

Various limitations of this study should be considered. We found a low annual eGFR decline of 0.82 mL/min/1.73 m^2^, which may seem low but it is however similar to what is reported in other healthcare utilization cohorts^26^. Furthermore, this is a CKD 3–5 cohort derived from a healthcare utilization database, and the indications for calcium and creatinine testing rendered a population selection of mainly elderly individuals. This old age may also be partially responsible for the overall low mean annual eGFR decline^27,28^. We also found a mortality rate of 35%, exceeding the total number of events of RRT (10%). However, it is broadly accepted that rates of death exceed those of RRT, especially in older age groups. This has been previously described in other healthcare cohorts^26,29^. Moreover, the association between serum calcium at baseline and subsequent annual eGFR decline was assumed to be linear and this is hard to confirm definitively. However, we performed trend analyses and showed that a linear assumption for the studied association seems justifiable. Another limitation is that our real-world healthcare utilization nature limits our capacity to have a full set of covariates (they are available only if the physician ordered the test), and we used multiple imputation to test as a sensitivity analysis the impact of correcting for iPTH and dipstick albuminuria. Multiple imputation is a preferred method independent of the proportion of missingness, if two assumptions are met: the number of observations should be sufficient and missing data should be reasonably related to observed patient characteristics (missing at random or MAR)^30^. We believe that both assumptions are easily met in our study. Further, it is uncertain if albuminuria can be regarded a confounder or, instead, to be within the causal pathway, and that is why we regard this as sensitivity analysis. A final limitation is that we did not have laboratory information on urine albumin/creatinine ratio, FGF23 levels, ionized calcium, 25(OH)D3 levels or HbA1c levels. Considering the above, the uncertainty of the results should be kept in mind.

The recently updated KDIGO guidelines on CKD-MBD management emphasize the need for optimal monitoring of serum calcium in CKD stages 3–5, based on the presence and magnitude of abnormalities^31,32^. In addition, guidelines suggest avoiding hypercalcemia, and state that mild and asymptomatic hypocalcemia can be tolerated in order to avoid inappropriate calcium loading. Furthermore, rising PTH levels or above the upper limit should be evaluated for hypocalcemia or vitamin D deficiency. However, solid evidence what the appropriate level is for lower serum calcium is lacking. We propose that low calcium levels may be interpreted as a proxy for increased FGF23 or deficiency of vitamin D in clinical practice. If the lower serum calcium levels are indeed indicative for either vitamin D deficiency or FGF23 excess, interventions should aim to restore this disorder. Possible interventions should not involve calcium supplementation, but most likely instead the prescription of native vitamin D, as also advised in current KDIGO guidelines, especially when a deficiency is established or suspected based on calcium levels^32,33^. In order to investigate the causal role of serum calcium in CKD progression, a RCT with vitamin D therapy would be required. The use of calcium supplements in CKD patients raises concerns about safety, given the attention to the plausible risks of calcium overload^33,34^. However, partly because of this, the potential role of lower serum calcium in CKD progression may not be recognized.

In summary, we showed in our large CKD 3–5 cohort that lower serum calcium, already within the normal reference range, was associated with a subsequent faster kidney function decline in individuals with CKD stages 3b, 4 and 5 not requiring dialysis. This association remained after adjustment for various confounders. Lower serum calcium may be indicative for vitamin D deficiency. If confirmed, these results may have clinical implications for disease-preventive strategies and emphasize the need to better delineate the role of calcium in the course of disease.

## Methods

### Study design, setting and study subjects

The Stockholm CREAtinine Measurements (SCREAM) project is a healthcare utilization cohort from the sole healthcare provider in the region of Stockholm, Sweden (Stockholm County Council), described elsewhere in more detail^35,36^. SCREAM collected healthcare information on all Stockholm residents over the age of 18 years with a valid personal identification number and who had a measurement of serum creatinine undertaken in in- or outpatient care during 2006–2011. For these individuals, all standard laboratory tests performed during the period were retrieved. The dataset was then linked to regional and national administrative databases with complete information on demographic data, healthcare utilization, diagnoses, validated end stage renal disease outcomes, vital status and pharmacy-dispensed medicines. The institutional review board for use of de-identified data at Karolinska Institutet, Stockholm, Sweden and the Swedish National Board of Welfare approved the study. Because data is de-identified, no informed consent is necessary according to Swedish ethical rules.

From this healthcare utilization database, we constructed a cohort study with participants having CKD stages 3–5. The index date was the date of the first eGFR test available per adult participant at study entry. We then selected all those participants with eGFR <60 mL/min/1.73 m^2^ after entry to construct a cohort of individuals classified as having CKD stages 3–5. Of those, we selected participants that had a concurrent measurement of serum calcium (defined as a serum calcium test taken at index date of up to 90 days before index date). For the purpose of this study (progression of CKD), we excluded individuals with prior renal replacement therapy, as ascertained by linkage with the Swedish Renal Registry. We then derived information on comorbid history, concomitant medication use and laboratory values from the other linked data sources. Because this is a real-world healthcare database, the availability of other laboratory tests at the time of index date depends on healthcare use and physicians’ ordering of the test.

### Biochemical assessments and study covariates

All blood and urine laboratory tests were performed as part of a healthcare encounter. Biochemical assessments were performed routinely by three different laboratories that provide services to the region (Aleris, Unilabs and Karolinska). Inter- as well as intra-laboratory variation is considered minimal, with the three laboratories being frequently audited for quality and harmonization by the national Government-funded organisation EQUALIS (www.equalis.se). We considered only laboratory tests performed in the outpatient setting as they reflect stable medical conditions. Serum creatinine measurements were standardized to isotope dilution mass spectrometry. The eGFR was estimated using the Chronic Kidney Disease Epidemiology Collaboration (CKD-EPI) formula, taking into account age, sex and serum creatinine. Data on ethnicity were not available by law, but we expected the misclassification of eGFR to be minimal, given the vast majority of residents in the Stockholm region is Caucasian. We extracted information of any concomitant testing, if available, of serum calcium, serum intact parathyroid hormone (iPTH), serum phosphorus, serum hemoglobin (Hb), serum albumin and dipstick albuminuria. To maximize the inclusion of data, we considered laboratory tests performed at index date or the closest to index date and up to 90 days before. Serum calcium levels were corrected for serum albumin by the conventional Payne’s formula: corrected calcium = measured calcium (mg/dL) +0.8 × (4- serum albumin [g/dL])^37^.

Other study covariates were considered as follows: age was defined as age at index date and analyzed continuously. Comorbid history was calculated from ICD-10 codes issued during 5 years prior to index date, with the exception of Diabetes Mellitus history, which was ascertained over the preceding 25 years because of its non-transient nature and long-term effects. Charlson Comorbidity index domains were used for identification of major diseases^38^. According to these domains, cardiovascular disease was defined as acute myocardial infarction, congestive heart failure, peripheral vascular disease and cerebrovascular disease; Diabetes mellitus was considered as the composite of diabetes with and without complications. Hypertension was defined by *(a)* relevant ICD-10 codes (ICD-10 I10-I15) and *(b)* pharmacy dispensation of antihypertensive medication (ATC codes for diuretics C03, RAAS inhibitors C09, C03DA, beta-blockers C07 and calcium channel blockers C08). Information on drug-dispensations comes from linkage with the Swedish Prescribed Drug Registry, collecting information on all prescription drugs dispensed at Swedish pharmacies. For the purpose of this study, repeated dispensations of calcium supplements (ATC code A12AA04, A12AA06, A12AA12, A12AX), phosphate binders (ATC code V03AE), active vitamin D analogues (ATC code A11CC04, A11CC03, H05BX02, H05BX03) and diuretics (ATC code C03) were extracted. Intake of medication at study inclusion considered any dispensation in the 3 months prior to the baseline measurement.

### Study exposure

The study exposure was serum calcium. To test the hypothesis that the association between serum calcium and CKD progression depends on CKD stage, analyses were stratified according to CKD stages at baseline. CKD staging 3–5 was based on KDIGO criteria (i.e. stage 3 eGFR 30–59 mL/min/1.73 m^2^, stage 4 eGFR 15–29 mL/min/1.73 m^2^ and stage 5 eGFR <15 mL/min/1.73 m^2^)^2^. CKD stage 3 was further subdivided in stage 3a (eGFR 45–59 mL/min/1.73 m^2^) and stage 3b (i.e. eGFR 30–44 mL/min/1.73 m^2^)^39,40^.

### Study outcome

The study outcome was the change in annual eGFR decline counted from the baseline. The rate of decline was defined as the absolute change in eGFR per year. This was calculated from all available consecutive eGFR measurements as performed in healthcare. In this analysis, patients were censored if they emigrated from the region, initiated renal replacement therapy, died or reached end of the observation period, which was December 31, 2011, whichever came first. Information on vital status was obtained via linkage with the Swedish Population Registry, and information on emigration from the region was supplied by the Healthcare provider records cross-matched with the regional censoring office.

### Statistical analyses

Categorical variables are presented as percentage of total; continuous variables are presented as mean values with standard deviation (SD) or median with interquartile range, depending on the  distribution. Baseline characteristics are presented for the total study population and stratified by CKD stage. P-values are two-tailed, and P < 0.05 was considered statistically significant. All analyses were performed with SPSS version 23.0.

Missing values were imputed with multiple imputation methods using a fully conditional specification with 10 repetitions^41–43^. Besides potential confounders, all available baseline variables and follow-up time were used for imputation. Follow-up time was logarithmically transformed; age and baseline eGFR values were square root transformed before entering in the imputation model. Estimates and standard deviations were calculated in each imputation set and pooled into one overall estimate and standard deviation according to Rubin’s rules^44,45^. Multiple imputation is the preferred method compared to complete case analysis in case of missing data^30,41,46,47^. Complete case analysis will lead to biased estimates and loss of power. The preference for multiple imputation is independent of the proportion of missingness up to 90%^30^.

Linear mixed models (LMM) with random intercept and slope were used to estimate the change in the annual rate of kidney function decline associated with one unit (1 mg/dl) increase in baseline calcium. This model examines how serial eGFR measurements depended on baseline serum calcium. Results are expressed as regression coefficients and 95% CIs. Results are reported as the absolute change in annual rate of decline in kidney function that can be attributed to a unit increase in calcium at baseline. A negative change indicates a greater decline due to calcium increase; and a positive change indicates less decline^48^. Multivariable analyses were used to adjust for potential baseline confounders. In a first model, we adjusted for age, sex, presence of DM, CVD, hypertension, serum albumin and hemoglobin. In a second model, we further adjusted for serum phosphate, active vitamin D therapy and calcium supplements. We did not adjust for iPTH in the primary analysis because iPTH lies in the causal pathway of the hypothesis hereby tested^49^. Instead, iPTH adjustment was considered in a sensitivity analysis (see below). We neither adjusted for phosphate binder use, since these frequently contain calcium, as such acting as calcium supplements^50^. LMM analyses were stratified by CKD stage. To investigate a potential dose-response relationship between baseline serum calcium and eGFR decline across baseline eGFR levels, we included an interaction term with baseline eGFR in the complete dataset combining all CKD stages. For increasing baseline eGFR (i.e. lower CKD stage), the coefficient for this interaction term estimates the additional change in kidney function decline associated with a unit (i.e. mg/dL) increase in baseline serum calcium.

To validate the robustness of our findings, several additional sensitivity analyses were performed. Analyses were repeated (1) adjusting for baseline eGFR levels; (2) in the subgroup of patients using vitamin D supplementation; (3) after adjustment for imputed albuminuria and iPTH. The additional adjustment for albuminuria was performed, given that active vitamin D deficiency contributes to progressive kidney function decline via albuminuria^51^; (4) adjusting for diuretics (ATC code C03) and hypertension (ICD-10 I10–15), separately; (5) categorizing calcium by quintiles of distribution. This was done to assess the potential of non-linear trends in the association between calcium and CKD progression; (6) using uncorrected serum calcium as the exposure, because the precision of this corrected value to predict the “gold standard” free (ionized) calcium is limited and because albumin might be a determinant of the outcome of interest^52,53^; (7) selecting only participants whose corrected serum calcium was within the normal reference range (i.e. 8.6–10.2 mg/dL); (8) selecting only participants with at least 3 eGFR tests available during follow up; (9) complete-case analysis (without multiple imputation); and (10) finally we used Cox proportional-hazards regression analysis for the assessment of the association between baseline serum calcium levels and subsequent risk of either a sustained GFR decline of more than 30% or the risk of RRT. These were considered secondary outcomes, because dichotomization of the outcome leads to loss of information and power.

### Data availability

All data generated or analysed during this study are included in this published article (and its Supplementary Information files). The datasets used and/or analysed during the current study are available from the corresponding author on reasonable request.

## Electronic supplementary material

Supplementary Information

## References

[1] Termorshuizen F (2004). Relative contribution of residual renal function and different measures of adequacy to survival in hemodialysis patients: an analysis of the Netherlands Cooperative Study on the Adequacy of Dialysis (NECOSAD)−2. J Am Soc Nephrol..

[2] KDIGO (2013). 2012 Clinical Practice Guideline for the Evaluation and Management of Chronic Kidney Disease. Kidney Int..

[3] Palmer SC (2011). Serum levels of phosphorus, parathyroid hormone, and calcium and risks of death and cardiovascular disease in individuals with chronic kidney disease: a systematic review and meta-analysis. JAMA..

[4] O’Neill WC (2016). Targeting serum calcium in chronic kidney disease and end-stage renal disease: is normal too high?. Kidney Int..

[5] Bellasi A (2011). Chronic Kidney Disease Progression and Outcome According to Serum Phosphorus in Mild-to-Moderate Kidney Dysfunction. Clin J Am Soc Nephrol..

[6] Caravaca F (2011). Relationship between serum phosphorus and the progression of advanced chronic kidney disease. Nefrologia..

[7] Voormolen N (2007). High plasma phosphate as a risk factor for decline in renal function and mortality in pre-dialysis patients. Nephrol Dial Transplant..

[8] Schwarz S, Trivedi BK, Kalantar-Zadeh K, Kovesdy CP (2006). Association of disorders in mineral metabolism with progression of chronic kidney disease. Clin J Am Soc Nephrol..

[9] Isakova T (2011). Fibroblast growth factor 23 is elevated before parathyroid hormone and phosphate in chronic kidney disease. Kidney Int..

[10] Lim LM (2014). Low serum calcium is associated with poor renal outcomes in chronic kidney disease stages 3-4 patients. BMC Nephrol..

[11] Ossareh S (2011). Vascular calcification in chronic kidney disease: mechanisms and clinical implications. Iran J Kidney Dis..

[12] Levin A (2007). Prevalence of abnormal serum vitamin D, PTH, calcium, and phosphorus in patients with chronic kidney disease: results of the study to evaluate early kidney disease. Kidney Int..

[13] Slatopolsky E, Robson AM, Elkan I, Bricker NS (1968). Control of phosphate excretion in uremic man. J Clin Invest..

[14] Siyam FF, Klachko DM (2013). What is hypercalcemia? The importance of fasting samples. Cardiorenal Med..

[15] Taylor JM (2017). R. T. Urinary Calcium Excretion and Risk of Chronic Kidney Disease in the General Population. KI Reports..

[16] Shigematsu T, Caverzasio J, Bonjour JP (1993). Parathyroid removal prevents the progression of chronic renal failure induced by high protein diet. Kidney Int..

[17] de Boer IH (2011). Serum 25-hydroxyvitamin D and change in estimated glomerular filtration rate. Clin J Am Soc Nephrol..

[18] Mehrotra R (2008). Hypovitaminosis D in chronic kidney disease. Clin J Am Soc Nephrol..

[19] Melamed ML (2009). 25-hydroxyvitamin D levels, race, and the progression of kidney disease. J Am Soc Nephrol..

[20] Shimada T (2004). FGF-23 is a potent regulator of vitamin D metabolism and phosphate homeostasis. J Bone Miner Res..

[21] Yamazaki Y (2008). Anti-FGF23 neutralizing antibodies show the physiological role and structural features of FGF23. J Bone Miner Res..

[22] Fliser D (2007). Fibroblast growth factor 23 (FGF23) predicts progression of chronic kidney disease: the Mild to Moderate Kidney Disease (MMKD) Study. J Am Soc Nephrol..

[23] Titan SM (2011). FGF-23 as a predictor of renal outcome in diabetic nephropathy. Clin J Am Soc Nephrol..

[24] Saito H (2005). Circulating FGF-23 is regulated by 1alpha, 25-dihydroxyvitamin D3 and phosphorus *in vivo*. J Biol Chem..

[25] Jean G, Souberbielle JC, Chazot C (2009). Monthly cholecalciferol administration in haemodialysis patients: a simple and efficient strategy for vitamin D supplementation. Nephrol Dial Transplant..

[26] Eriksen BO, Ingebretsen OC (2006). The progression of chronic kidney disease: a 10-year population-based study of the effects of gender and age. Kidney Int..

[27] O’Hare AM (2007). Age affects outcomes in chronic kidney disease. J Am Soc Nephrol..

[28] De Nicola L (2015). Independent Role of Underlying Kidney Disease on Renal Prognosis of Patients with Chronic Kidney Disease under Nephrology Care. PLoS One..

[29] Keith DS, Nichols GA, Gullion CM, Brown JB, Smith DH (2004). Longitudinal follow-up and outcomes among a population with chronic kidney disease in a large managed care organization. Arch Intern Med..

[30] Janssen KJ (2010). Missing covariate data in medical research: to impute is better than to ignore. J Clin Epidemiol..

[31] KDIGO Clinical Practice Guideline for the Diagnosis, Evaluation, Prevention, and Treatment of Chronic Kidney Disease-Mineral and Bone Disorder (CKD-MBD). *Kidney Int*. **7** [Suppl]: S1–S59 (2017). 10.1038/ki.2009.188 19644521

[32] Ketteler M (2017). Executive summary of the 2017 KDIGO Chronic Kidney Disease-Mineral and Bone Disorder (CKD-MBD) Guideline Update: what’s changed and why it matters. Kidney Int..

[33] Goodman WG (2000). Coronary-artery calcification in young adults with end-stage renal disease who are undergoing dialysis. N Engl J Med..

[34] Barry EL (2014). Calcium supplementation increases blood creatinine concentration in a randomized controlled trial. PLoS One..

[35] Runesson B, G. A., *et al*. The Stockholm CREAtinine Measurements (SCREAM) Project; protocol overview and regional representativeness. *Clin Kidney J*. 1–9 (2015). 10.1093/ckj/sfv117 PMC4720196 26798472

[36] Klatte, D. C. F. *et al*. Association Between Proton Pump Inhibitor Use and Risk of Progression of Chronic Kidney Disease. *Gastroenterology*. (2017). 10.1053/j.gastro.2017.05.046 28583827

[37] Payne RB, Little AJ, Williams RB, Milner JR (1973). Interpretation of serum calcium in patients with abnormal serum proteins. Br Med J..

[38] Charlson ME, Pompei P, Ales KL, MacKenzie CR (1987). A new method of classifying prognostic comorbidity in longitudinal studies: development and validation. J Chronic Dis..

[39] Go AS, Chertow GM, Fan D, McCulloch CE, Hsu CY (2004). Chronic kidney disease and the risks of death, cardiovascular events, and hospitalization. N Engl J Med..

[40] Chronic Kidney Disease Prognosis C (2010). Association of estimated glomerular filtration rate and albuminuria with all-cause and cardiovascular mortality in general population cohorts: a collaborative meta-analysis. Lancet..

[41] de Goeij MC (2013). Multiple imputation: dealing with missing data. Nephrol Dial Transplant..

[42] Schafer JL (1999). Multiple imputation: a primer. Stat Methods Med Res..

[43] van Buuren S (2007). Multiple imputation of discrete and continuous data by fully conditional specification. Stat Methods Med Res..

[44] Kenward MG, Carpenter J (2007). Multiple imputation: current perspectives. Stat Methods Med Res..

[45] Rubin, D. Multiple Imputation for Nonresponse in Surveys (John Wiley & Sons, 1987).

[46] Donders AR, van der Heijden GJ, Stijnen T, Moons KG (2006). Review: a gentle introduction to imputation of missing values. J Clin Epidemiol..

[47] Mackinnon A (2010). The use and reporting of multiple imputation in medical research - a review. J Intern Med..

[48] Fitzmaurice, G. M., Laird, N. M. & Ware, J. H. Applied LongitudinalAnalysis. John Wiley & Sons, Inc., Hoboken, NJ. (2011).

[49] Malberti F, Farina M, Imbasciati E (1999). The PTH-calcium curve and the set point of calcium in primary and secondary hyperparathyroidism. Nephrol Dial Transplant..

[50] Hill KM (2013). Oral calcium carbonate affects calcium but not phosphorus balance in stage 3-4 chronic kidney disease. Kidney Int..

[51] Sonneveld R (2016). 1,25-Vitamin D3 Deficiency Induces Albuminuria. Am J Pathol..

[52] Goransson LG, Skadberg O, Bergrem H (2005). Albumin-corrected or ionized calcium in renal failure? What to measure?. Nephrol Dial Transplant..

[53] Gauci C (2008). Pitfalls of measuring total blood calcium in patients with CKD. J Am Soc Nephrol..

