# Supplementary material for: Lower serum calcium is independently associated with CKD progression
Source: Sci Rep. 2018 Mar 26;8:5148. doi: 10.1038/s41598-018-23500-5 (PMC5980097; doi:10.1038/s41598-018-23500-5)
Supplement: Supplementary file 1 — Supplementary Information [file 41598_2018_23500_MOESM1_ESM.pdf]

Supplement to: Lower serum calcium is independently associated with CKD progression

Cynthia J. Janmaat, MD[1], Merel van Diepen, PhD[1], Alessandro Gasparini, Stat[2], Marie Evans, MD, PhD[2], Abdul Rashid Qureshi, MD, PhD[2], Johan Ärnlöv, MD, PhD[2], Peter Barany, MD, PhD[2], Carl-Gustaf Elinder, MD, PhD[2,3], Joris I. Rotmans, MD, PhD[4], Marc Vervloet, MD[5], Friedo W. Dekker, PhD[1], Juan Jesus Carrero, PhD[2,6]

Affiliations:
[1] Department of Clinical Epidemiology, Leiden University Medical Center, Leiden, The Netherlands
[2] Department of Clinical Science, Karolinska Institutet, Stockholm, Sweden
[3] Public Healthcare Services committee, Stockholm County Council, Stockholm, Sweden
[4] Department of Nephrology, Leiden University Medical Center, Leiden, The Netherlands
[5] Department of Nephrology and Institute for Cardiovascular Research VU, VU University Medical Center, Amsterdam, The Netherlands
[6] Department of Medical Epidemiology and Biostatistics, Karolinska Institutet, Stockholm, Sweden

Corresponding author:
Cynthia J. Janmaat
Department of Clinical Epidemiology
Leiden University Medical Center
PO Box 9600
2300 RC Leiden
The Netherlands
Phone +31 71 526 6539
Fax +31 71 5266994
Email: c.j.janmaat@lumc.nl

Table of Contents

	Page
**Supplementary Table S1.** Association between baseline corrected serum calcium with the subsequent rate of kidney function decline – additional adjustment for baseline eGFR	3
**Supplementary Table S2.** Association between baseline corrected serum calcium and the subsequent rate of kidney function decline (95%-CI) in the subgroup of patients with vitamin D supplementation at baseline	3
**Supplementary Table S3a.** Association between baseline corrected serum calcium with the subsequent rate of kidney function decline – additional adjustment for albuminuria and iPTH at baseline	4
**Supplementary Table S3b.** Multiplicative interaction tests between baseline corrected serum calcium and baseline eGFR in association with kidney function decline – additional adjustment for albuminuria and iPTH at baseline	4
**Supplementary Table S4.** Association between baseline corrected serum calcium with the subsequent rate of kidney function decline (95% CI) – model 1 of the main analysis adjusted for hypertension and diuretics, separately	4
**Supplementary Table S5.** Association between quintiles of baseline corrected serum calcium and subsequent rate of decline in kidney function	5
**Supplementary Table S6a.** Association between baseline serum calcium (not albumin-corrected) with subsequent rate of decline in kidney function	6
**Supplementary Table S6b.** Multiplicative interaction tests between baseline serum calcium (not albumin-corrected) and baseline eGFR in association with subsequent kidney function decline	6
**Supplementary Table S7a.** Association between baseline corrected serum calcium and the subsequent rate of kidney function decline (95%-CI) in patients with baseline serum calcium within normal range (8.6-10.2 mg/dL)	7
**Supplementary Table S7b.** Multiplicative interaction tests between baseline corrected serum calcium and baseline eGFR in its association with subsequent kidney function decline (95%-CI) in patients with baseline serum calcium within normal range (8.6-10.2 mg/dL)	7
**Supplementary Table S8a.** Association between baseline corrected serum calcium and the subsequent rate of kidney function decline (95%-CI) in patients with at least 3 eGFR tests available	8
**Supplementary Table S8b.** Multiplicative interaction tests between baseline corrected serum calcium and baseline eGFR in its association with subsequent kidney function decline (95%-CI) in patients with at least 3 eGFR tests available	8
**Supplementary Table S9a.** Association between baseline corrected serum calcium and subsequent kidney function decline - not imputed data	9
**Supplementary Table S9b.** Multiplicative interaction tests between baseline corrected serum calcium and baseline eGFR in its association with subsequent kidney function decline - not imputed data	9
**Supplementary Table S10.** Cox proportional-hazards regression analysis of the association between baseline corrected serum calcium and the subsequent risk of sustained decline in GFR of >30% (95%-CI)	10
**Supplementary Table S11.** Cox proportional-hazards regression analysis of the association between baseline corrected serum calcium and the subsequent risk of RRT(95%-CI)	10

Supplementary Table S1. Association between baseline corrected serum calcium with the subsequent rate of kidney function decline (95% CI) – additional adjustment for baseline eGFR

	CKD 3a (*n*=9286)	P*	CKD 3b (*n*=4190)	P*	CKD 4 (*n*=1784)	P*	CKD 5 (*n*=495)	P*
Change in eGFR decline per mg/dL increase in for albumin corrected calcium at baseline (negative = greater decline; positive = less decline)[a]								
Model 3	-0.027 (-0.488; 0.542)	0.92	0.353 (0.034; 0.671)	0.03	0.295(-0.062;0.653)	0.11	0.704 (0.363; 1.044)	<0.001

[a] In mL/min/1.73 m^2 per year
Model 3 adjusted for covariates in model 2 plus eGFR values at baseline
*P-value for difference in the change in the rate of kidney function decline with one unit increase in calcium

Supplementary Table S2. Association between baseline corrected serum calcium and the subsequent rate of kidney function decline (95%-CI) in the subgroup of patients with vitamin D supplementation at baseline

	CKD 3b (*n*=175)	P*	CKD 4 (*n*=366)	P*	CKD 5 (*n*=275)	P*
Change in eGFR decline per each mg/dL higher albumin-corrected calcium (negative = extra decline)[a]						
Raw data	-0.366 (-1.290; 0.559)	0.44	0.436 (-0.127; 0.999)	0.13	0.678 (0.311; 1.046)	<0.001
Model 1	-0.310 (-1.280; 0.661)	0.53	0.371 (-0.217; 0.959)	0.22	0.469 (0.068; 0.869)	0.02
Model 2	-0.309 (-1.286; 0.667)	0.54	0.375 (-0.215; 0.965)	0.21	0.446 (0.039; 0.853)	0.03
Model 3	-0.283 (-1.265; 0.700)	0.57	0.365 (-0.227; 0.956)	0.23	0.403 (-0.003; 0.810)	0.05

[a] In mL/min/1.73 m^2 per year.
Model 1 adjusted for age, sex, blood pressure, DM, CVD, serum albumin and hemoglobin
Model 2 adjusted for covariates in model 1 plus serum phosphorus and calcium supplements
Model 3 adjusted for covariates in model 2 plus active vitamin D therapy
*P-value for difference in the change in the rate of kidney function decline with one unit higher serum calcium

Supplementary Table S3a. Association between baseline corrected serum calcium with the subsequent rate of kidney function decline (95% CI) – additional adjustment for both albuminuria and iPTH at baseline

	CKD 3a (*n*=9286)	P*	CKD 3b (*n*=4190)	P*	CKD 4 (*n*=1784)	P*	CKD 5 (*n*=495)	P*
Change in eGFR decline per mg/dL increase in for albumin corrected calcium at baseline (negative = greater decline; positive = less decline)[a]								
Model 3	-0.009 (-0.278; 0.260)	0.95	0.392 (0.075; 0.710)	0.02	0.343 (-0.017; 0.703)	0.06	0.683 (0.357; 1.008)	<0.001

[a] In mL/min/1.73 m^2 per year
Model 3 adjusted for covariates in model 2 plus albuminuria and iPTH at baseline
*P-value for difference in the change in the rate of kidney function decline with one unit higher serum calcium

Supplementary Table S3b. Multiplicative interaction tests between baseline corrected serum calcium and baseline eGFR in association with kidney function decline 95% CI) – additional adjustment for both albuminuria and iPTH at baseline

	All patients (n=15755)	P*
Additional change in eGFR decline per each mg/dL higher albumin-corrected calcium for each mL/min/1.73m^2 higher unit of eGFR (negative = smaller effect)		
Model 3	-0.019 (-0.030; 0.008)	0.001

Model 3 adjusted for covariates in model 2 plus albuminuria and iPTH at baseline
*P-value for difference in the change in the rate of kidney function decline with one unit higher serum calcium

Supplementary Table S4. Association between baseline corrected serum calcium with the subsequent rate of kidney function decline (95% CI) – model 1 of the main analysis adjusted for hypertension and diuretics, separately

	CKD 3a (*n*=9286)	P*	CKD 3b (*n*=4190)	P*	CKD 4 (*n*=1784)	P*	CKD 5 (*n*=495)	P*
Change in eGFR decline per mg/dL increase in for albumin corrected calcium at baseline (negative = greater decline; positive = less decline)[a]								
Model 1a	-0.032 (-0.288; 0.222)	0.81	0.384 (0.066; 0.702)	0.02	0.355 (-0.002; 0.711)	0.05	0.675 (0.350; 0.999)	<0.001
Model 1b	-0.014 (-0.217; 0.189)	0.92	0.387 (0.070; 0.704)	0.02	0.353 (-0.003; 0.710)	0.05	0.674 (0.351; 0.998)	<0.001

[a] In mL/min/1.73 m^2 per year
Model 1a adjusted for age, sex, DM, CVD, serum albumin, hemoglobin and blood pressure (defined as presence hypertension
Model 1b adjusted for age, sex, DM, CVD, serum albumin, hemoglobin and diuretics
*P-value for difference in the change in the rate of kidney function decline with one unit increase in calcium

Supplementary Table S5. Association between quintiles of baseline corrected serum calcium and subsequent rate of decline in kidney function (95% CI)

	CKD 3a (n=9286)	P*	CKD 3b (n=4190)	P*	CKD 4 (n=1784)	P*	CKD 5 (n=495)	P*
Change in eGFR decline per mg/dL increase in albumin corrected calcium at baseline (negative = greater decline; positive = less decline)[a]								
Raw data								
Q1 (<9.2)	0.153 (-0.245; 0.552)	0.45	-0.605 (-1.121; -0.899)	0.02	-0.857 (-1.419; -0.296)	0.003	-0.960 (-1.837; -0.082)	0.03
Q2 (9.2-9.4)	0.087 (-0.311; 0.485)	0.67	-0.702 (-1.217; -0.187)	0.01	-0.490 (-1.419; 0.230)	0.11	-0.862 (-1.927; 0.203)	0.11
Q3 (9.4-9.6)	-0.094 (-0.489; 0.299)	0.64	-0.660 (-1.164; -0.157)	0.01	-0.826 (-1.392; -0.261)	0.004	-0.729 (-1.832; 0.373)	0.20
Q4 (9.6-9.9)	-0.116 (-0.509; 0.277)	0.56	-0.447 (-0.949; 0.055)	0.08	-0.531 (-1.111; 0.049)	0.07	-0.272 (-1.227; 0.683)	0.58
Q5 (>9.9)	reference		reference		reference		reference	
Model 1								
Q1 (<9.2)	0.047 (-0.340; 0.434)	0.82	-0.336 (-0.853; 0.181)	0.20	-0.740 (-1.328; -0.153)	0.01	-0.985 (-1.888; -0.083)	0.03
Q2 (9.2-9.4)	-0.011 (-0.409; 0.388)	0.96	-0.569 (-1.076; -0.061)	0.03	-0.393 (-0.997; 0.209)	0.20	-0.877 (-1.957; 0.204)	0.11
Q3 (9.4-9.6)	-0.181 (-0.570; 0.207)	0.37	-0.559 (-1.052; -0.066)	0.03	-0.729 (-1.301; -0.156)	0.01	-0.587 (-1.687; 0.512)	0.29
Q4 (9.6-9.9)	-0.205 (-0.594; 0.184)	0.30	-0.345 (-0.835; 0.145)	0.17	-0.506 (-1.086; 0.075)	0.09	-0.104 (-1.053; 0.845)	0.83
Q5 (>9.9)	reference		reference		reference		reference	
Model 2								
Q1 (<9.2)	0.060 (-0.345; 0.464)	0.77	-0.340 (-0.856; 0.176)	0.20	-0.749 (-1.337; -0.161)	0.01	-0.953 (-1.876; 0.031)	0.04
Q2 (9.2-9.4)	-0.004 (-0.403;0.395)	0.99	-0.567 (-1.072; -0.059)	0.03	-0.383 (-0.989; 0.221)	0.21	-0.869 (-1.963; 0.225)	0.12
Q3 (9.4-9.6)	-0.171 (-0.562;0.221)	0.39	-0.564 (-1.056; -0.072)	0.03	-0.722 (-1.296; -0.148)	0.01	-0.584 (-1.707; 0.538)	0.31
Q4 (9.6-9.9)	-0.200 (-0.589;0.189)	0.31	-0.356 (-0.846; 0.134)	0.15	-0.504 (-1.088; 0.081)	0.09	-0.103 (-1.063; 0.858)	0.83
Q5 (>9.9)	reference		reference		reference		reference	

[a] In mL/min/1.73 m^2 per year
Model 1 adjusted for age, sex, blood pressure, DM, CVD serum albumin and hemoglobin
Model 2 adjusted for covariates in model 1 plus serum phosphorus, active vitamin D therapy and calcium supplements
*P-value for difference in the change in the rate of kidney function decline with one unit increase in calcium

Supplementary Table S6a. Association between baseline serum calcium (not albumin-corrected) with subsequent rate of decline in kidney function (95% CI)

	CKD 3a (*n*=9286)	P*	CKD 3b (*n*=4190)	P*	CKD 4 (*n*=1784)	P*	CKD 5 (*n*=495)	P*
Change in eGFR decline per mg/dL increase in calcium at baseline (negative = greater decline; positive = less decline)[a]								
Raw data	0.299 (0.053; 0.544)	0.02	0.732 (0.445; 1.020)	<0.001	0.455 (0.130; 0.780)	0.01	0.638 (0.332; 0.945)	<0.001
Model 1	-0.003 (-0.251; 0.244)	0.98	0.390 (0.073; 0.708)	0.02	0.328 (-0.030; 0.686)	0.07	0.683 (0.359; 1.008)	<0.001
Model 2	-0.009 (-0.227; 0.260)	0.85	0.391 (0.074; 0.708)	0.02	0.344 (-0.015; 0.704)	0.06	0.682 (0.355; 1.009)	<0.001

[a] In mL/min/1.73 m^2 per year
Model 1 adjusted for age, sex, blood pressure, DM, CVD serum albumin and hemoglobin
Model 2 adjusted for covariates in model 1 plus serum phosphorus, active vitamin D therapy and calcium supplements
*P-value for difference in the change in the rate of kidney function decline with one unit higher serum calcium

Supplementary Table S6b. Multiplicative interaction tests between baseline serum calcium (not albumin-corrected) and baseline eGFR in association with subsequent kidney function decline (95% CI)

	All patients (n=15755)	P*
Additional change in eGFR decline per each mg/dL higher calcium for each mL/min/1.73m^2 higher unit of eGFR (negative = smaller effect)		
Raw data	-0.008 (-0.018; -0.003)	0.16
Model 1	-0.009 (-0.015; -0.004)	0.08
Model 2	-0.009 (-0.015; -0.004)	0.08

Model 1 adjusted for age, sex, blood pressure, DM, CVD serum albumin and hemoglobin
Model 2 adjusted for covariates in model 1 plus serum phosphorus, active vitamin D therapy and calcium supplements
*P-value for difference in the change in the rate of kidney function decline with one unit higher serum calcium

Supplementary Table S7a. Association between baseline corrected serum calcium and the subsequent rate of kidney function decline (95%-CI) in patients with baseline serum calcium within normal range (8.6-10.2 mg/dL)

	**CKD 3a (*n*=8663)**	**P***	**CKD 3b (*n*=3785)**	**P***	**CKD 4 (*n*=1577)**	**P***	**CKD 5 (*n*=386)**	**P***
Change in eGFR decline per each mg/dL higher albumin-corrected calcium (negative = extra decline)[a]								
Raw data	-0.302 (-0.681; 0.078)	0.12	0.547 (0.034; 1.055)	0.04	0.645 (0.120; 1.169)	0.02	0.649 (0.319; 0.978)	<0.001
Model 1	-0.229 (-0.614; 0.157)	0.25	0.286 (-0.224; 0.796)	0.27	0.546 (-0.003; 1.094)	0.05	0.693 (-0.164; 1.551)	0.11
Model 2	-0.233 (-0.620; 0.154)	0.24	0.278 (-0.231; 0.787)	0.29	0.541 (-0.009; 1.091)	0.05	0.630 (-0.246; 1.507)	0.16

[a] In mL/min/1.73 m^2 per year.

Model 1 adjusted for age, sex, blood pressure, DM, CVD, serum albumin and hemoglobin

Model 2 adjusted for covariates in model 1 plus serum phosphorus, active vitamin D therapy and calcium supplements

*P-value for difference in the change in the rate of kidney function decline with one unit higher serum calcium

Supplementary Table S7b. Multiplicative interaction tests between baseline corrected serum calcium and baseline eGFR in its association with subsequent kidney function decline (95%-CI) in patients with baseline serum calcium within normal range (8.6-10.2 mg/dL)

	**All patients (n=14411)**	**P***
Additional change in eGFR decline per each mg/dL higher albumin-corrected calcium for each mL/min/1.73m^2 higher unit of eGFR (negative = smaller effect)		
Raw data	-0.041 (-0.061; -0.022)	<0.001
Model 1	-0.039 (-0.058; -0.019)	<0.001
Model 2	-0.039 (-0.058; -0.019)	<0.001

Model 1 adjusted for age, sex, blood pressure, DM, CVD serum albumin and hemoglobin

Model 2 adjusted for covariates in model 1 plus serum phosphorus, active vitamin D therapy and calcium supplements

*P-value for difference in the change in the rate of kidney function decline with one unit higher serum calcium

Supplementary Table S8a. Association between baseline corrected serum calcium and the subsequent rate of kidney function decline (95%-CI) in patients with at least 3 eGFR test available

	CKD 3a (*n*=4786)	P*	CKD 3b (*n*=2426)	P*	CKD 4 (*n*=1220)	P*	CKD 5 (*n*=395)	P*
Change in eGFR decline per each mg/dL higher albumin-corrected calcium (negative = extra decline)[a]								
Raw data	-0.072 (-0.338; 0.194)	0.60	0.476 (0.156; 0.797)	0.004	0.409 (0.064; 0.754)	0.02	0.648 (0.322; 0.973)	<0.001
Model 1	0.016 (-0.230; 0.263)	0.12	0.359 (0.039; 0.678)	0.03	0.318 (-0.043; 0.678)	0.08	0.680 (0.356; 1.003)	<0.001
Model 2	0.010 (-0.260; 0.281)	0.94	0.360 (0.041; 0.679)	0.03	0.335 (-0.026; 0.696)	0.07	0.681 (0.355; 1.007)	<0.001

[a] In mL/min/1.73 m^2 per year.

Model 1 adjusted for age, sex, blood pressure, DM, CVD, serum albumin and hemoglobin

Model 2 adjusted for covariates in model 1 plus serum phosphorus, active vitamin D therapy and calcium supplements

*P-value for difference in the change in the rate of kidney function decline with one unit higher serum calcium

Supplementary Table S8b. Multiplicative interaction tests between baseline corrected serum calcium and baseline eGFR in its association with subsequent kidney function decline (95%-CI) in patients with at least 3 eGFR test available

	All patients (n=8827)	P*
Additional change in eGFR decline per each mg/dL higher albumin-corrected calcium for each mL/min/1.73m^2 higher unit of eGFR (negative = smaller effect)		
Raw data	-0.018 (-0.030; -0.007)	0.002
Model 1	-0.017 (-0.028; -0.005)	0.004
Model 2	-0.017 (-0.028; -0.005)	0.003

Model 1 adjusted for age, sex, blood pressure, DM, CVD serum albumin and hemoglobin

Model 2 adjusted for covariates in model 1 plus serum phosphorus, active vitamin D therapy and calcium supplements

*P-value for difference in the change in the rate of kidney function decline with one unit higher serum calcium

Supplementary Table S9a. Association between baseline corrected serum calcium and subsequent kidney function decline (95% CI) - not imputed data

	CKD 3a (*n*=9286)	P*	CKD 3b (*n*=4190)	P*	CKD 4 (*n*=1784)	P*	CKD 5 (*n*=495)	P*
Change in eGFR decline per mg/dL increase in for albumin corrected calcium at baseline (negative = greater decline; positive = less decline)[a]								
Raw data	-0.098 (-0.362; 0.165)	0.47	0.515 (0.196; 0.835)	0.002	0.428 (0.084; 0.772)	0.015	0.649 (0.319; 0.978)	<0.001
Model 1	-0.108 (-0.194; 0.410)	0.48	0.328 (-0.037; 0.692)	0.08	0.262 (-0.150;0.676)	0.21	0.630 (0.285; 0.974)	<0.001
Model 2	0.011 (-0.524; 0.546)	0.97	0.364 (-0.187; 0.915)	0.20	0.499 (0.036; 0.963)	0.04	0.355 (0.109; 0.601)	0.01

[a] In mL/min/1.73 m^2 per year
Model 1 adjusted for age, sex, blood pressure, DM, CVD serum albumin and hemoglobin
Model 2 adjusted for covariates in model 1 plus serum phosphorus, active vitamin D therapy and calcium supplements
*P-value for difference in the change in the rate of kidney function decline with one unit higher serum calcium

Supplementary Table S9b. Multiplicative interaction tests between baseline corrected serum calcium and baseline eGFR in association with subsequent kidney function decline (95% CI) - not imputed data

	All patients (n=15755)	P*
Additional change in eGFR decline per each mg/dL higher albumin-corrected calcium for each mL/min/1.73m^2 higher unit of eGFR (negative = smaller effect)		
Raw data	-0.021 (-0.032; -0.009)	<0.001
Model 1	-0.014 (-0.027; -0.002)	0.02
Model 2	-0.012 (-0.027; 0.003)	0.11

Model 1 adjusted for age, sex, blood pressure, DM, CVD serum albumin and hemoglobin
Model 2 adjusted for covariates in model 1 plus serum phosphorus, active vitamin D therapy and calcium supplements
*P-value for difference in the change in the rate of kidney function decline with one unit higher serum calcium

Supplementary Table S10. Cox proportional-hazards regression analysis of the association between baseline corrected serum calcium and the subsequent risk of sustained decline in GFR of >30% (95%-CI)

	**CKD 3a (*n*=9286)**	**P***	**CKD 3b (*n*=4190)**	**P***	**CKD 4 (*n*=1784)**	**P***	**CKD 5 (*n*=495)**	**P***
**Number events**	**547**		**451**		**438**		**158**	
HR for having event per each mg/dL higher albumin-corrected calcium[a]								
Raw data	1.19 (0.98; 1.44)	0.08	0.90 (0.74; 1.10)	0.31	0.75 (0.62; 0.90)	0.003	0.79 (0.64; 0.97)	0.03
Model 1	1.06 (0.87; 1.29)	0.53	0.98 (0.81; 1.19)	0.86	0.83 (0.68; 1.02)	0.08	0.84 (0.68; 1.03)	0.09
Model 2	1.06 (0.87; 1.29)	0.54	0.98 (0.81; 1.18)	0.84	0.84 (0.68; 1.03)	0.09	0.82 (0.67; 1.01)	0.06

[a] In mL/min/1.73 m^2 per year.
Model 1 adjusted for age, sex, blood pressure, DM, CVD, serum albumin and hemoglobin
Model 2 adjusted for covariates in model 1 plus serum phosphorus, active vitamin D therapy and calcium supplements
*P-value for difference in the change in the rate of kidney function decline with one unit higher serum calcium

Supplementary Table S11. Cox proportional-hazards regression analysis of the association between baseline corrected serum calcium and the subsequent risk of RRT (95%-CI)

	**CKD 3a (*n*=9286)**	**P***	**CKD 3b (*n*=4190)**	**P***	**CKD 4 (*n*=1784)**	**P***	**CKD 5 (*n*=495)**	**P***
**Number events**	**29**		**89**		**265**		**246**	
HR for having event per each mg/dL higher albumin-corrected calcium[a]								
Raw data	0.89 (0.37; 2.14)	0.80	0.65 (0.40; 1.03)	0.07	0.62 (0.48; 0.79)	<0.001	0.92 (0.79; 1.07)	0.25
Model 1	0.81 (0.32; 2.00)	0.64	0.79 (0.51; 1.24)	0.31	0.74 (0.57; 0.95)	0.02	0.90 (0.78; 1.04)	0.16
Model 2	0.81 (0.33; 2.02)	0.65	0.79 (0.50; 1.22)	0.28	0.73 (0.57; 0.94)	0.02	0.87 (0.75; 1.00)	0.06

[a] In mL/min/1.73 m^2 per year.
Model 1 adjusted for baseline eGFR, age, sex, blood pressure, DM, CVD, serum albumin and hemoglobin
Model 2 adjusted for covariates in model 1 plus serum phosphorus, active vitamin D therapy and calcium supplements
*P-value for difference in the change in the rate of kidney function decline with one unit higher serum calcium
